# Paper-Based Analytical Devices for the Rapid and Direct Electrochemical Detection of Hydrogen Peroxide in Tomato Leaves Inoculated with *Botrytis cinerea*

**DOI:** 10.3390/s20195512

**Published:** 2020-09-26

**Authors:** Lijun Sun, Yu Pan, Jin Wu, Danyang Zhao, Meiqi Hui, Suqin Zhu, Xinyu Zhu, Dayong Li, Fengming Song, Cankui Zhang

**Affiliations:** 1 Department of Biological Sciences, School of Life Sciences, Nantong University, 9 Seyuan Rd, Nantong 226019, Jiangsu, China; 1809310004@stmail.ntu.edu.cn (Y.P.); 1509011058@stmail.ntu.edu.cn (J.W.); 1609011042@stmail.ntu.edu.cn (D.Z.); 1609011038@stmail.ntu.edu.cn (M.H.); zhusuqin@ntu.edu.cn (S.Z.); zhuxinyu@ntu.edu.cn (X.Z.); 2 National Key Laboratory for Rice Biology, Institute of Biotechnology, Zhejiang University, Hangzhou 310029, Zhejiang, China; dyli@zju.edu.cn (D.L.); fmsong@zju.edu.cn (F.S.); 3 Department of Agronomy and Center for Plant Biology, Purdue University, West Lafayette, IN 47907, USA; ckzhang@purdue.edu

**Keywords:** hydrogen peroxide, electrochemical detection, paper-based analytical devices, nano-gold modified indium tin oxide electrodes, tomato, *Botrytis cinerea*

## Abstract

Hydrogen peroxide (H_2_O_2_) is an important signaling molecule and plays key roles in multiple plant physiological processes. The rapid and direct monitoring of H_2_O_2_ could improve our understanding of its regulatory mechanisms in plants. In this study, we developed a paper-based analytical device consisting of a disposable nano-gold modified indium tin oxide working electrode to provide a platform for the rapid and direct detection of H_2_O_2_. The total analytical time was dramatically shortened to be approximate 3 min due to the avoidance of the time-consuming and complex treatment of plant samples. In addition, the amount of plant samples required was less than 3 mg in our approach. We used this system to monitor the concentrations of H_2_O_2_ in tomato leaves infected by *Botrytis* *cinerea* within 24 h. Our results showed that the concentration of H_2_O_2_ in tomato leaves was increased in the initial phase, peaked at 1.5 μmol gFW^−1^ at 6 h, and then decreased. The production trend of H_2_O_2_ in tomato leaves inoculated with *Botrytis* *cinerea* detected with our approach is similar to the 3,3-diaminobenzidine staining method. Taken together, our study offers a rapid and direct approach for the detection of H_2_O_2_, which will not only pave the way for the further investigation of the regulation mechanisms of H_2_O_2_ in plants, but also promote the development of precision agriculture technology.

## 1. Introduction

Hydrogen peroxide (H_2_O_2_), a simple molecule discovered by Louis Jacques Denard 100 years ago, is one of the primary reactive oxygen species (ROS) in plants [1,2,3]. Compared to other ROS, such as superoxide anion radicals (O2•−) and hydroxyl radicals (OH•), H_2_O_2_ is relatively stable, less reactive, and electrically neutral. These characteristics of H_2_O_2_ enable the molecule to translocate cell membranes and reach cell locations that are distant from the site of its formation [1,2,3]. H_2_O_2_ can be produced via various metabolic pathways in plant organelles, including chloroplasts, mitochondria, and peroxisomes [4,5,6]. H_2_O_2_ plays key roles in multiple plant physiological processes, including participating in responses to biotic stresses [1,2,7,8]. The possible mechanisms of the H_2_O_2_ response to pathogen infection have been documented, including the modification of the cell wall, the signal transduction pathway, programmed cell death, and system-acquired resistance [7,8]. When plants are infected by pathogens, H_2_O_2_ is produced and accumulated at the infected sites (oxidative burst), which can trigger programmed cell death to prevent the further spread of the pathogen [9]. H_2_O_2_ also crosstalks with plant hormones such as salicylic acid, jasmonates, and auxin to regulate the plant defense reactions [10,11,12,13,14]. For example, the concentration of H_2_O_2_ could be increased under salicylic acid-binding protein inhabitable catalase activity [15,16], while salicylic acid can increase the quantity of H_2_O_2_, which induces the expression of defense-related genes associated with systemic-acquired resistance [16].

Although studies on the roles of H_2_O_2_ in biotic stress responses have made great advances [1,2,7,8], there is still no direct evidence for the biosynthesis sites, synthesis initiation times, and transport of H_2_O_2_ during the interaction between plant and pathogen, which greatly limits the capacity to determine the distribution of H_2_O_2_ and its dynamic change in plants. Therefore, the rapid monitoring of H_2_O_2_ is essential in understanding its regulatory mechanisms in plants. Currently, the detection of H_2_O_2_ is carried out mostly via techniques such as titration, spectroscopy colorimetry, chromatography, chemiluminescence, and fluorescence [17,18,19,20,21,22]. Such methods have limited success due to the long duration required to prepare samples. For example, plant samples need to be separated and ground before detection. The complex and time-consuming pre-treatments may lead to the loss or alteration of H_2_O_2_ concentrations or forms, which makes it a challenging task to accurately obtain the concentration of H_2_O_2_.

Electrochemical methods provide a convenient means for the detection of H_2_O_2_ due to their relative ease of operation, high selectivity, and sensitivity in molecule detection and quantification [23]. For the electrochemical detection of H_2_O_2_ in plants, only a few reports involving microelectrode sensors have been described to detect the H_2_O_2_ in plant cells [24,25] or leaves [26,27] in situ and in real time. For example, a dual-function platinum disc microelectrode sensor was used for the in situ monitoring of H_2_O_2_ produced in *Agave tequilana* leaves after inoculation with their endophytic bacteria [27]. Paper-based electroanalytical devices also provide a potential alternative approach for the detection of H_2_O_2_ in plants in situ and in real time. In our previous reports, we have integrated paper-based electrochemical devices with MWCNTS-Nafion-modified carbon tape electrodes directly on tomato leaves for the in situ determination of salicylic acid concentrations with the sample volume of several microliters [28]. The concentrations of both indole-3-acetic acid and salicylic acid could be rapidly quantified in plant samples to the microgram level [29].

In the present study, an electrochemical detection platform consisting of a paper-based analysis device, a disposable nano-gold-modified indium tin oxide (ITO) working electrode, and an electrochemical workstation for the detection of H_2_O_2_ in plants were developed. Compared with traditional methods for the detection of H_2_O_2_, our study provided a rapid and direct approach for the detection of H_2_O_2_ in plants, which will help to further investigate the regulating mechanisms of H_2_O_2_ in plants. In addition, assisted by a portable electrochemical workstation, our system allows the detection of H_2_O_2_ in plants grown in the field, which will promote the development of precision agriculture technology.

## 2. Materials and Methods

### 2.1. Chemicals and Materials

Hydrogen tetrachloroaurate (III) trihydrate (HAuCl_4_•3H_2_O), abscisic acid, indole-3-acetic acid, salicylic acid, jasmonic acid, methyl jasmonate, and ascorbic acid were purchased from Sigma (St. Louis, MO, USA). The H_2_O_2_ (30% wt. in water) was purchased from Shanghai Chemical Reagent Company. All the other chemical reagents were of analytical grade. The ITO conductive glass (40.64 × 35.56 × 0.11 cm STN, 10 ohm) was obtained from Nanbo Display Technology Co., Ltd. (Shenzhen, Guangdong, China). The Whatman No. 1 qualitative filter paper was obtained from GE Healthcare Bio-Sciences (Pittsburgh, PA, USA). The Harris Uni-CoreTM (Tip ID 4.0 mm) Miltex^®^ was purchased from Ted Pella Inc. (Redding, CA, USA). The tomato seeds (Shanghai 906, one-generation hybrid) were purchased from Lintong Changfeng Vegetable Breeding Farm in Xi’an City. The H_2_O_2_ was obtained from Shanghai Zhanyun Chemical Co. Ltd. (Shanghai, China). Double-distilled water was used in all the experiments.

### 2.2. Material Preparation

HAuCl_4_•3H_2_O (10 g/L, 34 μL) was added to 1 mmol/L of KCl (200 μL), then 3766 μL of double-distilled water was added to prepare 4 mL of 0.25 mmol/L HAuCl_4_•3H_2_O, and stored in a refrigerator at 4 °C for later use in electroplating. The H_2_O_2_ (30% wt. in water) was diluted using 0.1 M of phosphate buffered solution (pH 7.0) for detection. Tomato plants were grown in a mixture of perlite: vermiculite: plant ash (1:6:2) in a growth room at 22 °C under a 16 h light and 8 h dark regime. For analysis of the concentration of H_2_O_2_ produced in response to pathogen infection, four-week-old tomato leaves were sprayed with spore suspensions or a buffer as mock inoculation (control). Briefly, spores were collected in 1% maltose buffer from 10-day-old *Botrytis cinerea* cultures grown on 2 × V8 agar (36% V8 juice, 0.2% CaCO_3_, 2% agar) by passing through two layers of cheesecloth with a spore density of 1 × 10^5^ spores mL^-1^. The inoculated plants were covered with a transparent plastic film and maintained in a growth chamber with conditions similar to plant growth conditions. Leaves from at least four individual plants were used in each experiment.

### 2.3. Paper-Based Electroanalytical Devices and Electrochemical Detection

**Scheme 1:**
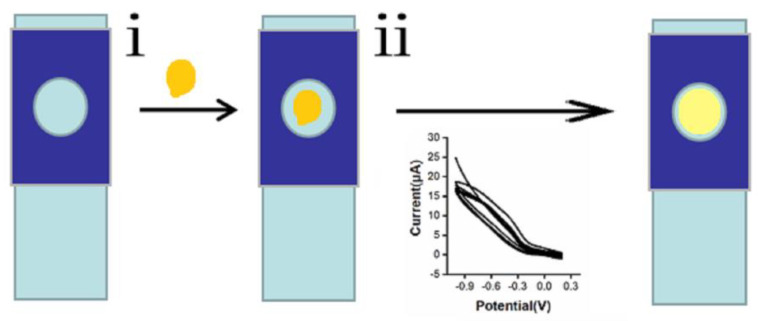
Schematic diagram of the nano-gold modified indium tin oxide (ITO) electrode. (**i**) A total of 10 μL of HAuCl_4_•3H_2_O solution was dropped on the conductive surfaces of the ITO glasses (4 mm holes), (**ii**) the HAuCl_4_•3H_2_O solution was electroplated on the conductive surfaces of the ITO glasses by cyclic voltammetry.

For the nano-gold-modified ITO electrode preparation, the ITO glass was cut into 20 × 8 mm pieces and washed using acetone and ethanol ultrasonically for 10 min. The washed ITO glasses were cleaned repeatedly with double-distilled water to remove acetone and ethanol. The cleaned ITO glasses were dried in an oven at 50 °C. As showed in the Scheme 1, 10 μL of HAuCl_4_•3H_2_O solution was dropped on the conductive surfaces of the ITO glasses adhered with a layer of perforated (4 mm) transparent tape. An Ag/AgCl electrode and a platinum wire electrode were used as a reference electrode and counter electrode, respectively. Then, the HAuCl_4_•3H_2_O solution was electroplated on the conductive surfaces of the ITO glasses (4-mm holes) by cyclic voltammetry with the parameters −1–0.2 V potential range, 0.1 V/s scanning speed, 10 scanning segments, 0.001-V sampling intervals, and 2 s standing time. The modified electrode was named a nano-gold-modified ITO electrode.

**Scheme 2:**
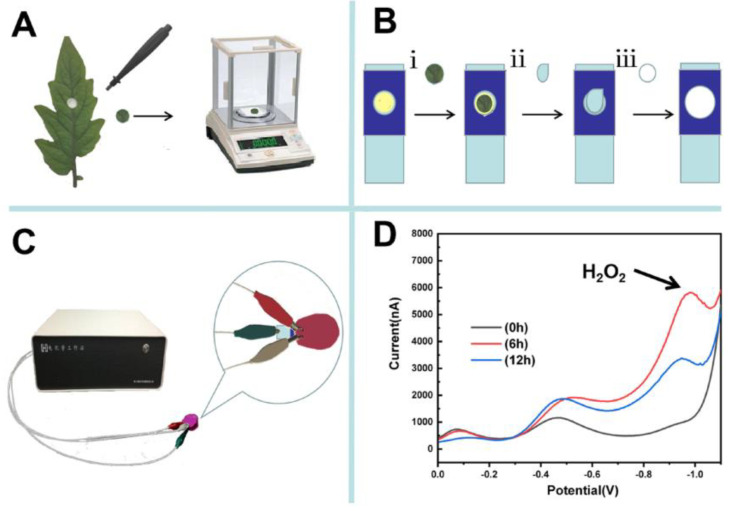
Schematic diagram of the rapid and direct detection of H_2_O_2_ in tomato leaves. (**A**) Leaves of tomato inoculated with spores of *Botrytis cinerea* or buffer solution were retrieved by the Miltex^®^ Biopsy Punch. The collected tomato leaf samples with diameters of 4 mm were weighted. (**B**) The collected tomato leaf samples were kept on the surface of a nano-gold-modified ITO electrode, then 10 μL of buffer was dropped on the tissue surface and a piece of filter paper was used to cover the electrode surface. (**C**) Detection of H_2_O_2_ in the tomato leaf samples with a CHI 1240C electrochemical station. (**D**) The typical differential pulse voltammetry detection curves of H_2_O_2_ in the tomato leaf samples inoculated with spores of *Botrytis cinerea*.

For electrochemical detection, tomato leaves inoculated with spore suspensions of *Botrytis cinerea* or buffer solution (Mock) were obtained using a Miltex Biopsy Punch with a diameter of 4 mm (Scheme 2A). The circular forms of the retrieved leaf samples were weighed and placed on the surface of a nano-gold-modified ITO electrode, then 10 μL of phosphate buffer solution with a pH of 7.0 was dropped on the electrode, and the electrode surface was covered with a piece of filter paper (Scheme 2B). H_2_O_2_ was detected in tomato leaf using a CHI 1240C electrochemical workstation (CH Instruments Inc., Austin, TX, USA). A three-electrode system consisting of a modified nano-gold electrode (working electrode), an Ag/AgCl electrode (reference electrode), and a platinum wire electrode (counter electrode) was used (Scheme 2C). The H_2_O_2_ was detected using differential pulse voltammetry with a −1.2–0 V potential range, a potential increment of 0.005 V, an amplitude of 0.05 V, a pulse width of 0.2 s, a sampling width of 0.067 s, a pulse period of 0.5 s, and a standing time of 2 s. Before each test, the counter electrode and the reference electrode were washed with double-distilled water thoroughly. The H_2_O_2_ concentrations in tomato leaves infected with *Botrytis cinerea* were determined directly and rapidly (Scheme 2D).

### 2.4. Diaminobenzidine Staining

To validate our approach for the detection of H_2_O_2_ in tomato leaves, 3-3′ diaminobenzidine (DAB) staining was performed according to the methods described previously [30]. Briefly, tomato leaves were collected from inoculated plants 0, 1, and 6 h after inoculation with *Botrytis cinerea* spores and dipped into DAB solution (0.5 mg/mL, pH 3.8) for 8 h in the dark at room temperature. The DAB-treated leaves were placed into 95% ethanol at 80 °C for 30 min to remove chlorophyll. Subsequently, the leaves were maintained in 60% glycerol and the accumulation of H_2_O_2_ was visualized using a digital camera.

## 3. Results and Discussion

**Figure 1:**
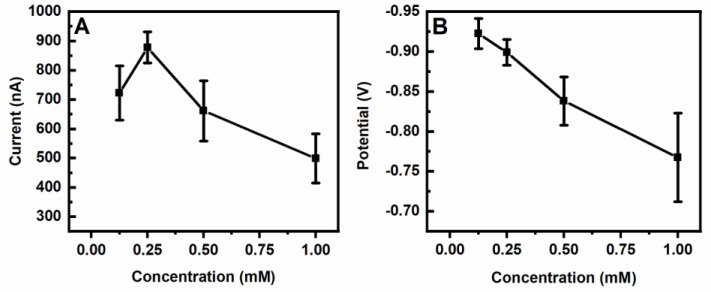
Dependence of the differential pulse voltammetry peak heights (**A**) and potential (**B**) of H_2_O_2_ (200 μM) on the ITO electrodes modified with different concentrations of HAuCl_4_•3H_2_O by electroplating. The average values and standard deviations were obtained based on 6 results.

**Figure 2:**
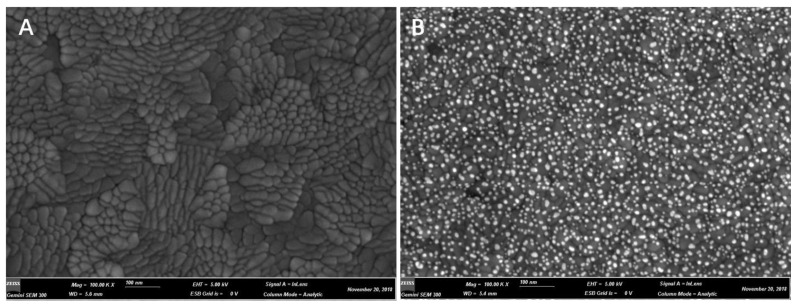
The scanning electron microscope images of the bare ITO electrode (**A**) and the ITO electrode modified with 0.25 mM (**B**) by electroplating.

Since the nano-gold-modified ITO electrodes was fabricated by electroplating HAuCl_4_•3H_2_O on the surface of the ITO electrodes, we investigated the influence of different concentrations of HAuCl_4_•3H_2_O at the ITO electrodes on the electrochemical responses of 200 μM of H_2_O_2_. We observed that the 0.25 mM of HAuCl_4_•3H_2_O-electroplated ITO electrodes exhibited higher electrochemical responses to the H_2_O_2_ (Figure 1A). Notably, there were no electrochemical responses to H_2_O_2_ on the bare ITO electrodes. The potential of H_2_O_2_ further increased from −0.925 V to −0.775 V when the concentration of HAuCl_4_•3H_2_O was increased (Figure 1B). In addition, the potential of H_2_O_2_ on the 0.25 mM of HAuCl_4_•3H_2_O-modified ITO electrodes was more stable. The surface characterizations of the bare ITO electrode and the 0.25mM of HAuCl_4_•3H_2_O-electroplated ITO electrodes were observed using a Hitachi S-3400 II scanning electron microscope. Compared to the bare ITO electrode, the 5~10 nm gold nanoparticles homogeneously distributed throughout the surface of the ITO electrode (Figure 2).

**Figure 3:**
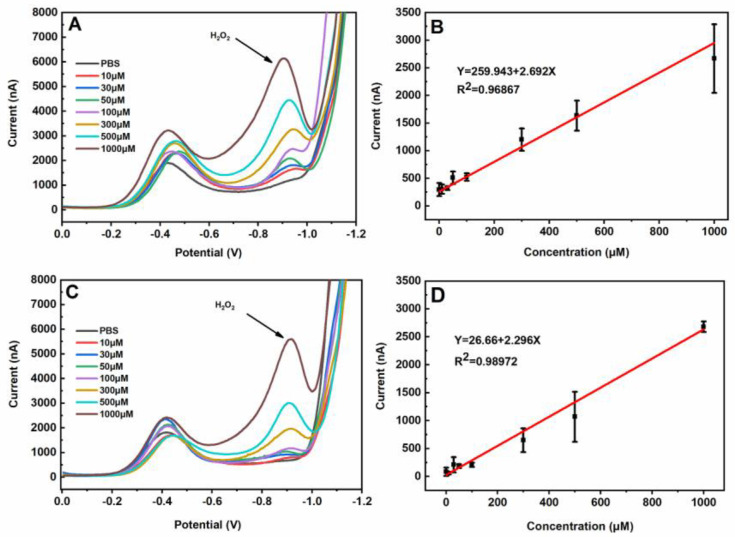
The differential pulse voltammetry curves of H_2_O_2_ with different concentrations (0~1000 μM) on the nano-gold-modified ITO electrodes (**A**) and the calibration curve between the differential pulse voltammetry peak currents and the H_2_O_2_ concentrations in the air (**B**), the differential pulse voltammetry curves of H_2_O_2_ with different concentrations (0~1000 μM) on the nano-gold-modified ITO electrodes (**C**), and the calibration curve between the differential pulse voltammetry peak currents and the H_2_O_2_ concentrations in the N_2_ atmosphere (**D**). The average values and standard deviations were obtained based on 6 replicates.

In the present study, H_2_O_2_ was quantified and verified based on the differential pulse voltammetry peak heights and potentials. In order to study the influence of the air on the H_2_O_2_ detection, prior to each experiment a stream of highly pure nitrogen was gently blown inside a plastic bag with the detection device to maintain the nitrogen atmosphere for at least 20 min. Figure 3 illustrates differential pulse voltammetry curves of H_2_O_2_ with various concentrations at the nano-gold-modified ITO electrodes in the air or nitrogen atmosphere. There were two peaks at approximately −0.4 and −0.9 V potentials. The peaks at −0.9 V further increased when the H_2_O_2_ concentration was increased, while the peaks at −0.4 V were irregular (Figure 3A,C). The oxidation peak currents of H_2_O_2_ displayed a linear response to the concentrations of H_2_O_2_ from 10 to 1000 μM in the air or nitrogen atmosphere (Figure 3B,D). The linear relationship between the peak current and the concentration was Y = 2.692X + 259.943 (R^2^ = 0.9687) in the air atmosphere and Y = 2.296X + 26.66 (R^2^ = 0.9897) in the nitrogen atmosphere (X: concentration of H_2_O_2_; Y: peak current magnitude). There were no obvious differences except for the base currents between the detection of H_2_O_2_ in the air and the nitrogen atmosphere. Considering the importance of the rapid detection of H_2_O_2_ from the plant sample, a strategy for the direct detection of H_2_O_2_ in the air was selected.

**Figure 4:**
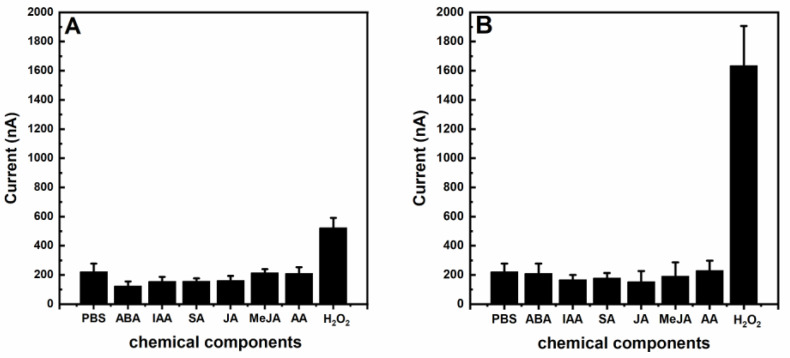
Electrochemical responses of signal molecules (abscisic acid (ABA), indole-3-acetic acid (IAA), salicylic acid (SA), jasmonic acid (JA), methyl jasmonate (MeJA), ascorbic acid (AA)) in plants with 100 μM (**A**) or 500 μM (**B**). The average values and standard deviations were obtained based on 6 replicates.

The detection limit and reproducibility are important parameters for the evaluation of sensor performance. The detection limit of our system was estimated to be 1 μM based on a signal-to-noise ratio of six. The reproducibility of the nano-gold-modified ITO electrode was estimated from the response to 100 μM of H_2_O_2_ using six different electrodes. The relative standard deviation was found to be 5.8%, indicating a good reproducibility for the sensor preparation. We investigated the potential interferences with the determination of H_2_O_2_ in the plants from plant signaling molecules, and found that there were no significant interferences in the presence of abscisic acid, indole-3-acetic acid, salicylic acid, jasmonic acid, methyl jasmonate, and ascorbic acid under 100 or 500 μM of H_2_O_2_ (Figure 4). The results indicated that nano-gold-modified ITO electrodes could facilitate the determination of H_2_O_2_ in plant samples. More importantly, the nano-gold-modified ITO electrodes could be fabricated as one-time-use disposable electrodes, which could avoid the contamination of electrodes. In addition, the fabrication of the nano-gold-modified ITO electrodes was feasible (less than five minutes to prepare one electrode) and suitable for mass production. Compared with previous reports, our strategy was superior regarding the simple work electrode preparation, good analytical performance, volume of buffer solution, and simple sample preparation for the H_2_O_2_ detection in plants (Table 1).

**Figure 5:**
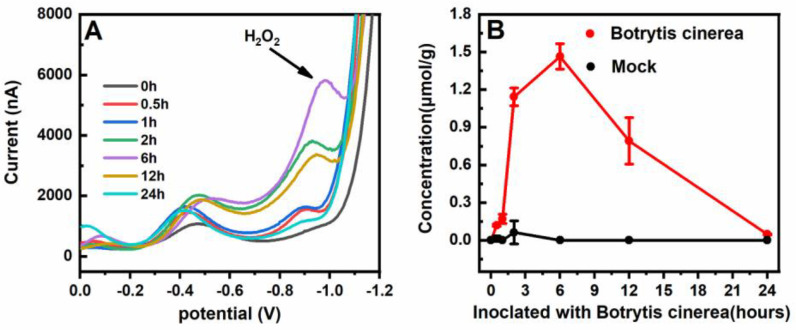
The typical differential pulse voltammetry detection curves (**A**) and contents of H_2_O_2_ (**B**) in tomato leaves at different times after being inoculated with *Botrytis cinerea*. The average values and standard deviations were obtained based on 12 replicates.

As a necrotrophic pathogen, *Botrytis cinerea* can colonize senescent or dead plant tissues and cause gray mold disease [34,35]. *Botrytis cinerea* infects over 200 dicot crop hosts such as tomato, grapes, and strawberry. Therefore, it could cause substantial economic losses [34,35]. In our experiments, typical disease symptoms—e.g., necrotic lesions—could be observed in tomato leaves inoculated with *Botrytis cinerea* spore suspensions at 3 dpi. Figure 5A illustrates the typical differential pulse voltammetry detection curves of H_2_O_2_ in the tomato leaf samples inoculated with *Botrytis cinerea* spores at different time points. H_2_O_2_ in the tomato leaf samples could be identified at a DPV peak potential of −0.90 V. It is necessary to emphasize that the total analytical time was dramatically shortened to approximately 3 min because our method avoided the time-consuming and complex treatment of tomato leaf samples. In addition, the amount of tomato leaf samples was less than 3 mg in our approach. The concentrations of H_2_O_2_ in the tomato leaf samples inoculated with *B. cinerea* increased at the initial phase and peaked at 1.5 μmol (gFW)^−1^ 6 h after inoculation, then decreased gradually until the presence of H_2_O_2_ could not be detected 24 h after inoculation (Figure 5B). In the normal tomato leaves, for comparison, almost no H_2_O_2_ could be observed at different time points (Figure 5B).

**Figure 6:**
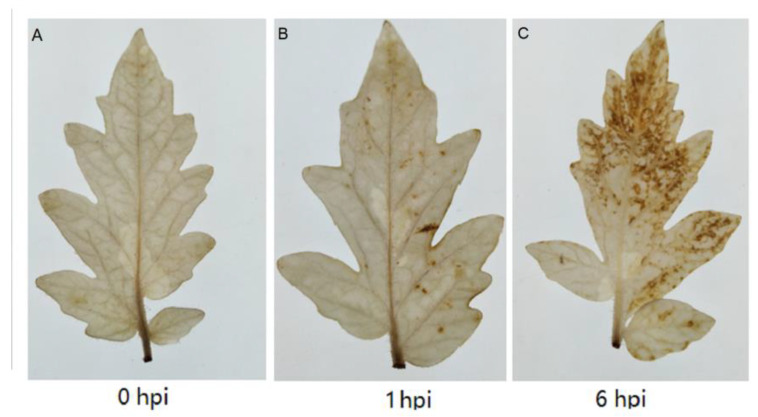
The detection of H_2_O_2_ production in tomato leaves after inoculation with *Botrytis cinerea* by DAB: 0 h (**A**), 1 h (**B**), 6 h (**C**).

In order to compare and evaluate the ability of our approach in detecting H_2_O_2_ production after tomato leaves were inoculated with *Botrytis cinerea*, DAB staining was also employed. As showed in Figure 6A, no significant accumulation of H_2_O_2_ was observed in tomato leaves 0 h after inoculation. One hour post-inoculation, a few spots due to the presence of H_2_O_2_ could be noticed (Figure 6B). Six hours after inoculation, much H_2_O_2_ could be visualized throughout the leaves. The production trend of H_2_O_2_ in tomato leaves inoculated with *Botrytis cinerea* based on DAB staining was similar in our method. Patykowski and Urbanek also reported that the H_2_O_2_ concentrations of tomato leaves inoculated with *Botrytis cinerea* increased as early as 5 h after inoculation, increasing to 0.2 μmol (gFW)^−1^ [36]. These results implied that our approach could offer a rapid and effective means of studying H_2_O_2_ dynamics in plants.

## 4. Conclusions

In the present study, we developed a platform using paper-based analytical devices coupled with nano-gold-modified ITO working electrodes for detecting H_2_O_2._ Our approach could detect H_2_O_2_ in plant samples at the milligram scale. In addition, the complex and time-consuming pre-treatment procedures required in conventional methods for the quantification of H_2_O_2_ in plant samples were avoided, and thus our method could determine the concentrations of H_2_O_2_ in plants more rapidly and directly. Using our approach, differentiable H_2_O_2_ concentrations were obtained in tomato leaves after infection with *Botrytis cinerea*. Our study presents a valid method that not only facilitates the investigation of the regulating mechanisms of H_2_O_2_ in plants but also promotes the development of precision agriculture technology.

## Figures and Tables

**Table 1:** List of the analytical performances of the electrochemical methods for the detection of H_2_O_2_ in plants.

Electrode System	Method	Applied Potential	Liner Range	Limit of Detection	Sample Application	Volume of Buffer Solution	Ref.
WE: Hb/SWCNTs/CFUMEs CE: Pt wire RE: Ag/AgCl _(sat. kCl)_	Amperometric	−0.1 V	4.9~0.405 mM	4 µM	Detection of aloe leaves in vivo	No description	[25]
WE: Platinum disc microelectrode RE: Silver expoxy coat, Ag/AgCl _(sat. kCl)_	Cyclic voltammogram	−1.0~0 V (−0.7 V)	100~100 mM	100 µM	Detection of agave tequilana leaves in vivo	No description	[26]
WE: HRP/colloidal Au/ pPA/Pt electrode CE: Pt wire RE: Saturated calomel electrode	Amperometric	0 V	0.42~1.5 mM	0.14 µM	Detection of the extraction solution of the plant leaves	5 mL BR	[31]
WE: HRP/Cys/AuNPs/ITO CE: platinum foil RE: Saturated calomel electrode	Amperometric	−0.15 V	8~3 mM	2 µM	Detection of the extraction solution of the plant leaves	10 mL PBS	[32]
WE: PtNPs/PAA/aSPCEs CE: Platinum wire RE: Ag/AgCl wire	Amperometric	0.1 V	0~300 µM	51.6 nM	Liquid oxygen solution for plants	10 mL PBS	[33]
WE: Au nanodots-ITO CE: Platinum wire RE: Ag/AgCl wire	Differential pulse voltammetry	−0.90 V	0~1 mM	1 µM	Detection of the direct tomato leaves (3 mg)	10 µl PBS	This study

Note: WE, work electrode; CE, counter electrode; RE, reference electrode; Hb/SWCNTs/CFUMEs, hemoglobin/single-walled carbon nanotubes/carbon fiber ultramicroelectrode; HRP/colloidal Au/ pPA/Pt electrode, horseradish peroxidase/colloidal Au/poly2,6-pyridinediamine/platinum wire electrode; HRP/Cys/AuNPs/ITO, horseradish peroxidase/L-Cysteine/gold nanoparticles/indium tin oxide; PtNPs/PAA/aSPCEs, poly (azure A)-platinum nanoparticles deposited on activated screen-printed carbon electrodes.
